# Development of a Smartphone-Based Balance Assessment System for Subjects with Stroke

**DOI:** 10.3390/s20010088

**Published:** 2019-12-22

**Authors:** You-Ruei Hou, Ya-Lan Chiu, Shang-Lin Chiang, Hui-Ya Chen, Wen-Hsu Sung

**Affiliations:** 1 Department of Physical Therapy and Assistive Technology, National Yang-Ming University, Taipei 112, Taiwan; yrhou.pt@gmail.com (Y.-R.H.); skywing0527@hotmail.com (Y.-L.C.); 2 Department of Physical Medicine and Rehabilitation, Tri-Service General Hospital, School of Medicine, National Defense Medical Center, Taipei 114, Taiwan; andyyy520@yahoo.com.tw; 3 Department of Physical Therapy, Chung Shan Medical University, Taichung 402, Taiwan; hychen@csmu.edu.tw

**Keywords:** smartphone, balance, stroke

## Abstract

Stroke is a cerebral artery disease that negatively affects activities of daily living (ADLs) and quality of life (QoL). Smartphones have demonstrated strong potential in assessing balance performance. However, such smartphone-based tools have thus far not been applied to stroke survivors. The purpose of this study was to develop a smartphone-based balance assessment system for subjects who have experienced strokes and evaluate the system feasibility. The smartphone-based balance assessment application was developed with Android Studio, and reliability and validity tests were conducted. The smartphone was used to record data using a built-in accelerometer and gyroscope, and increased changes represented greater instability. Six postures were tested for 30 s each. Ten healthy adults were recruited in the reliability test, and the intraclass correlation coefficient (ICC) was used to analyze the within-day and between-day reliabilities. Eight subjects with chronic stroke and eight healthy adults were recruited for the validity test, in which balance performance was compared to represent the application validity. The ICC values of the reliability tests were at least 0.76 (*p* = 0.00). The acceleration data exhibited no difference between individuals who have experienced stroke and healthy subjects; however, all six postures were found to differ significantly between the two groups in the gyroscope data. The study demonstrates that the smartphone application provides a convenient, reliable, and valid tool for the balance assessments of subjects who have experienced chronic stroke.

## 1. Introduction

Balance is a dominant factor impacting activities of daily living (ADLs). Insufficient balance ability may cause difficulties in sitting, standing, walking, and other functional activities; furthermore, loss of balance may decrease the QoL [1,2].

Individuals who have experienced stroke usually suffer from loss of ADLs and QoL performances, and importantly, they can also suffer loss of balance and injury. Stroke is a cerebrovascular disease that causes hemiplegia, muscle weakness, and sensory and motor deficits, which will result in stroke survivors facing a decrease in balance ability [3].

Balance ability has been defined as the ability to maintain the center of gravity (COG) within the base of support (BOS) with minimal postural sway. Static and dynamic balance are the two classifications for balance ability: the former represents the ability to maintain balance under static conditions, such as sitting and standing, and the latter represents the ability to maintain balance under dynamic conditions, such as walking and running [4,5,6,7].

Balance assessment is required to evaluate a person’s balance ability. Subjective and objective assessment are classified as follows: subjective assessments measure balance via standardized tests such as the Berg balance scale, the Romberg test, and the forward reach test; objective assessments evaluate balance by analyzing the data obtained from instruments, such as force plate balance, motion capture, and inertial measurement unit (IMU) balance assessments [8,9,10].

In clinical assessments, subjective data are usually collected. The Berg balance scale (BBS) is a 56-score-test including 14 static and dynamic functional tasks, and is regarded as one of the golden standards in assessing balance. The Romberg test observes the different standing performances between E/O and E/C conditions, and was first used for detecting vestibular issues; however, the Romberg test was also extended to assess balance ability, owing to the fact that a decrease in visual input may affect balance performance. The forward reach test is a dynamic balance test that evaluates how far a person can reach without losing balance: when a person is able to reach farther, their balance ability is considered to be superior [11,12,13].

Laboratory assessments often observe balance performance objectively, by using force plates, motion capture systems, and IMUs. Body-fixed IMUs, which include accelerometers and gyroscopes, approximate the COG when attached at the height of the center of mass (COM) [14]. Accelerometers collect acceleration data and can be used to compute various measures, such as the root mean square (RMS) from changes in acceleration, the total path length of the COG, and the total area of the COG pathway. Larger values of RMS, COG path length, and COG area indicate reduced balance performance. Gyroscopes collect angular velocity data, and can be used to compute changes in COG angular velocity and angular position. Larger values of COG angular velocity and position indicate reduced balance performance [12,13,14,15,16,17,18,19].

However, the existing balance assessments still exhibit certain limitations: subjective balance assessments are not precise, while objective balance assessments are not convenient. The precision of subjective tests relies on evaluator experience, and the balance assessment results also vary according to different testers. The majority of the objective assessment systems are neither portable nor easy to move; moreover, the evaluation procedure is complex, and a high time cost is involved.

Smartphones can be regarded as a solution for overcoming the limitations of existing balance assessments. Of course, mobility is one of the main features provided by smartphones. Furthermore, built-in sensors in every smartphone, including accelerometers and gyroscopes, are key to making smartphones capable of detecting balance. Therefore, by combining mobility and built-in IMUs, smartphones offer the potential to provide an objective and convenient balance assessment method [20,21].

Smartphones are already used to detect body movements, having been applied to investigate falls, measure postural sway, quantify gait performance, and assess balance ability [19]. However, balance-detection-related smartphone research mostly focus on healthy adults. Stroke survivors are facing balance issues due to the disease. Some balance evaluation postures (such as tandem stance) are not suitable for them, so a suitable application designed for stroke survivors is needed. Furthermore, smartphones have been applied to individuals who have experienced stroke and have been proven feasible in assessing balance. However, an application designed for assessing the balance ability of individuals who have experienced stroke does not yet exist. Therefore, the purpose of this study was to develop a smartphone application for assessing the balance performance of subjects who have experienced strokes and to evaluate the developed system’s feasibility.

## 2. Materials and Methods

### 2.1. System Development

Two major smartphone systems are currently available on the market: Android and iOS. We selected the Android system for our development environment owing to its 87.7% market share [22]. Android Studio 2.0 was the platform on which we programed the application, and the ASUS Zenfone 3 was the device selected for carrying out the smartphone-based balance assessment, owing to its low price and stable built-in sensors.

Three types of built-in smartphone sensors exist: motion sensors (accelerometers, gyroscopes, gravity sensors, and rotation vector sensors), position sensors (orientation sensors and magnetometers), and environmental sensors (barometers, thermometers, and photometers). We selected the accelerometer and the gyroscope to represent balance performance. The accelerometer collects linear acceleration, while the gyroscope gathers angular velocity. The sampling rate of the built-in accelerometer and gyroscope was set to 50 Hz [23].

### 2.2. Smartphone-Based Balance Assessment

Reductions in visual input and BOS have been proven to affect balance performance [24]. According to the above, we designed six standing postures with different difficulties for our balance assessment: shoulder-width-stance with eyes opened (SWS with E/O), shoulder-width-stance with eyes closed (SWS with E/C), feet-together-stance with eyes opened (FTS with E/O), feet-together stance with eyes closed (FTS with E/C), semi-tandem-stance with eyes opened (STS with E/O), and semi-tandem-stance with eyes closed (STS with E/C). For the semi-tandem-stance, participants who have experienced stroke were instructed to stand on their affected leg and take a full step forward with their unaffected leg, while healthy participants were instructed to take a full step forward with their dominant leg. Each posture was tested for 30 s following a 60 s break. The test was carried out by a physical therapist, with the instruction “please stand still with minimal body sway.”

The smartphone was fixed to the back of the trunk at the second sacrum spine level with a belt, representing the human body COG [19]. During the designed balance assessment, the smartphone collected accelerometer and gyroscope data, and then saved the data in the smartphone internal storage memory. Later, we used a computer to analyze the postural control ability with the data from the 10th to 20th second, to avoid the potential influence of the evaluator operating the smartphone at the beginning and the end of the assessment.

We calculated the combined changes in the acceleration vector from the anterior-poster and medial-lateral axes to represent the postural control ability: a greater acceleration change indicated more postural instability [25]. The calculation is completed as the following algorithm. *x* and y represents the acceleration of the *x*-axis and *y*-axis in m/s^2^; n is the total number of data samples of accelerometer:

(1) $$\frac{[\sqrt{{\left(x_{2}-x_{1}\right)}^{2}+{\left(y_{2}-y_{1}\right)}^{2}]}+[\sqrt{{\left(x_{3}-x_{2}\right)}^{2}+{\left(y_{3}-y_{2}\right)}^{2}]}+\cdots +[\sqrt{{\left(x_{n}-x_{n-1}\right)}^{2}+{\left(y_{n}-y_{n-1}\right)}^{2}]}}{n-1}$$

We monitored the changes in angular velocity from the pitch, roll, and yaw axes to determine the angle by which the body tilted during the test: a greater body tilting angle indicated lower postural control [26]. We used the following numerical integration approach that provides an approximation for angular position. The calculation is completed as the following algorithm, *a*, *b*, and *c* represent the angle tilted from the *x*-axis, *y*-axis, and *z*-axis; α, β, and γ represent the instantaneous angular velocity of the *x*-axis, *y*-axis, and *z*-axis in rad/s; *f* represents the sampling rate; 180 ÷ π changes the unit to degrees; n is the total number of data samples from the gyroscope:

(2) $$\left(a_{1}+a_{2}+\cdots +a_{n-1}+a_{n}\right)+\left(b_{1}+b_{2}+\cdots +b_{n-1}+b_{n}\right)+\left(c_{1}+c_{2}+\cdots +c_{n-1}+c_{n}\right)$$

$$a_{i}=\left|\alpha_{i}\right|÷f\times 180÷\pi$$

$$b_{i}=\left|\beta_{i}\right|÷f\times 180÷\pi$$

$$c_{i}=\left|\gamma_{i}\right|÷f\times 180÷\pi$$

*i* = 1, 2, 3, …, n

### 2.3. Reliability Test

The reliability was tested following the system development. Healthy adults were recruited for the reliability test, with the following inclusion criteria: aged between 20 and 65 years and free of any disease that may affect balance performance. An institutional review board of the Tri-Service hospital (Taipei, Taiwan) approved the clinical trial, and informed consent was obtained from each subject prior to beginning the experiment.

The within-day and between-day reliabilities were tested in our study. Following the first smartphone-based balance assessment, participants had a 30 min rest before undergoing the second assessment. The third assessment took place 48 h after the second assessment. The correlation of the first and second tests represented the within-day reliability; that of the first and third assessments indicated the between-day reliability. The ICC values were analyzed for the within-day and between-day reliability, and the statistical significance was set to *p* < 0.05.

### 2.4. Validity Test

Age-paired healthy adults and individuals who have experienced stroke participated in the validity test. Healthy adults were recruited with the following inclusion criteria: aged between 20 and 65 years and free of any disease that may affect balance performance. Subjects who have experienced strokes were recruited if they satisfied the following inclusion criteria: (1) aged between 20 and 65 years, (2) in at least stage 4 of the Brunnstrom states, (3) able to walk independently for more than 15 min indoors, (4) able to follow the instructions of testers, and (5) free of any disease or condition other than prior incidences of strokes that may affect balance performance.

Comparative and criterion validities were tested in this study. We first collected the age, height, and weight of the participants, and then assessed the balance ability with the BBS and smartphone-based balance test. In order to present the comparative validity test, we used an independent t-test to compare the balance performance of healthy adults and individuals who have experienced stroke measured by the smartphone. For the criterion validity test, we analyzed the Pearson’s correlation coefficient of the smartphone assessment and BBS results. The study design was approved by the institutional review board of the Tri-Service hospital, Taipei, Taiwan (No. 2-106-05-022). Patient consent was obtained.

The SPSS 20.0 software was used to analyze the reliability and validity test data, and the statistical significance was set to *p* < 0.05.

## 3. Results

### 3.1. Reliability Test

A total of 11 healthy adults participated in the reliability test, including five males and six females. The average participant age was 27.4 ± 3.2 years.

Table 1 displays the within-day and between-day ICC results of the accelerometer and gyroscope, obtained by means of the smartphone-based balance assessment application. The within-day and between-day ICC of the accelerometer were both over 0.75 (within-day: 0.904, *p* = 0.000; between-day: 0.764, *p* = 0.000), indicating excellent reliability. The within-day and between-day ICCs of the gyroscope were at least 0.857 (within-day: 0.897, *p* = 0.000; between-day: 0.857, *p* = 0.000), again indicating excellent reliability.

### 3.2. Validity Test

A total of 16 age-paired subjects recruited at the Tri-Service hospital (Taipei, Taiwan) participated in the validity test, including eight healthy adults and eight individuals who have experienced stroke. Every participant had completed all of the full 30 s trials for all conditions in our study. Table 2 provides the basic data of the participants. The table indicates that there were no significant differences in age (healthy group: 51.5 ± 9.0; stroke group: 52.3 ± 9.7, *p* = 0.770), height (healthy group: 165.3 ± 5.9; stroke group: 168.5 ± 9.1, *p* = 0.405), or weight (healthy group: 67.5 ± 10.2; stroke group: 72.6 ± 16.9, *p* = 0.364) between the groups; however, the healthy group significantly outperformed the stroke group in terms of the BBS score (healthy group: 56.0 ± 0.0; stroke group: 43.5 ± 4.1, *p* = 0.000).

Table 3 and Table 4 display the comparative validity results. Table 3 presents the comparison of the accelerometer data between the healthy adults and individuals who have experienced stroke, where a lower value represents superior balance performance. Although the healthy group performed slightly better than the stroke group in most of the smartphone balance tests (except for SWS with E/O), no significant statistical differences were observed in all six testing postures between the groups.

Table 4 provides a comparison of the gyroscope data between the healthy and stroke groups, where a lower value indicates superior balance performance. According to the table, the healthy adults performed significantly better than the subjects who have experienced stroke in all six testing postures.

Table 5 displays the criterion validity, illustrating the correlation between the smartphone-based balance assessment and BBS results. According to the table, none of the acceleration data were significantly correlated with the BBS. However, all of the gyroscope data were significantly correlated with the BBS, all with a high negative correlation.

## 4. Discussion

According to the reliability test, the developed application was proved to have excellent reliability. In the validity test, the accelerometer did not show the significant difference between the healthy group and the stroke group in all six testing postures; on the other hand, the gyroscope revealed different balance performances between the groups in all testing postures. Furthermore, none of the acceleration data were significantly correlated with the BBS score; instead, all of the gyroscope data were significantly correlated with the BBS score.

### 4.1. Difference between Existing Smartphone Applications

Two balance-assessment-related applications are available in the Google Play Store: YMED and Concussion Assessment & Response. The former uses built-in smartphone sensors to detect balance, while the latter evaluates balance ability by the user’s recording of the single-leg-stance test result [27,28]. The YMED and application we developed are both objective balance assessments, but the results displayed differ. YMED uses built-in sensors to draw the body movement in Cartesian coordinates, where increased movements drawn in the coordinates represent inferior balance ability. As there are no instructions in the YMED application, we do not know how to use it correctly, and it is not known for which type of balance ability or target group the application was designed. Concussion Assessment & Response is a subjective balance assessment application designed for healthy individuals and athletes. The users must record their balance performance themselves after taking the single-leg-stance test by following step-by-step instructions. According to the YMED and Concussion Assessment & Response features, these are not suitable for individuals who have experienced stroke. Therefore, we developed an application for patients who have experienced strokes; this application can assess balance ability objectively, display results with numbers, and evaluate balance with instructions.

### 4.2. Accelerometer and Gyroscope

The accelerometer and gyroscope play different roles in representing balance performance. The accelerometer collects the movement direction and acceleration, expressing the intensity of the body movement. The gyroscope gathers the rotational motion and velocity, indicating the degree to which the body sways following integration. In the comparative validity test, the accelerometer data of the healthy adults and individuals who have experienced stroke were not significantly different. The information gathered implied that the body movement intensity was similar for the healthy and stroke groups during the balance tests. Moreover, in the comparative validity test, the gyroscope data demonstrated that the healthy adults significantly outperformed the stroke participants. As we combined the results of both smartphone built-in sensors, we observed that the subjects who have experienced stroke swayed much more than the healthy adults during the balance tests, but the sway intensity was similar. In previous smartphone-related balance assessment studies, Mellone, Lee, and Patterson used only an accelerometer to obtain the balance parameters [29,30,31]. Although the abovementioned studies proved that the smartphone was feasible for detecting balance, certain results recorded by the accelerometers did not exhibit significant differences between the compared data. Based on the different characteristics of the accelerometer and gyroscope, we expected that the addition of the gyroscope data would provide a more complete balance assessment.

### 4.3. Feasibility of the Developed Application

In this study, reliability and validity tests were conducted to prove the feasibility of the developed smartphone-based balance assessment application. According to Table 1, the reliability test demonstrated that the application is stable. For the comparative validity test, Table 4 indicates that the healthy adults outperformed the individuals who have experienced stroke in all six balance testing postures. The results demonstrate that the quantified balance ability is valid. In the criterion validity test, we compared the developed application with the BBS, which is one of the golden standards in balance assessment. The gyroscope data were strongly related to the BBS score, which is a promising result. In summary, the application we developed can be regarded as feasible for assessing balance ability of individuals who have experienced stroke.

### 4.4. Limitation

A primary limitation of this study is the small sample size. The evaluator and smartphone were the same in the reliability test; our study would be more convincing if we included an equivalence reliability test to prove that the application is still reliable even when the users or smartphones differ.

The one specific smartphone used in the study is also a limitation. Not all of the smartphones on the market may not present the exact same reliability and validity as the results of our study. To deal with this probable problem, we chose a mid-price-range and mid-specification-range smartphone as the main device used in the study, and set the sampling rate at 50 Hz. Because the testing setting is not very critical, most smartphones on the market should have enough performance to execute the same setting and obtain a similar result. We will try another smartphone to verify the results in the future.

## 5. Further Investigation and Conclusions

In our system, the user can only compare the balance performance with their own records from the past. It is suggested that an age-related norm be built into the application, in order to provide better feedback to the users, as this would allow them to compare information with other users. Moreover, a dynamic balance assessment system is expected to be developed. Smartphones should be fixed on the back of the body when conducting balance assessments. In the future, hand-held evaluation will offer a more convenient means of using the application. Furthermore, the smartphone exhibits the potential to train balance ability; by utilizing the assessment results, we can create individualized balance training plans. In conclusion, we created a smartphone-based balance assessment system for subjects who have experienced stroke, and this system was proven to be feasible. In conclusion, the developed system is a reliable, valid, objective, and convenient balance assessment tool for assessing the static balance performances of patients who have experienced strokes.

## Figures and Tables

**Table 1:** Within-day and between-day reliability.

Sensors	Within-Day			Between-Day		
	ICC	95%CI	*p* Value	ICC	95%CI	*p* Value
ACC	0.904	0.844–0.941	0.00 **	0.764	0.615–0.856	0.00 **
GYR	0.897	0.797–0.948	0.00 **	0.857	0.732–0.924	0.00 **

** *p* < 0.01. ICC = intraclass correlation coefficient. CI = confidence interval. ACC = accelerometer data. GYR = gyroscope data.

**Table 2:** Basic data of the participants.

	Healthy Group (*n* = 8)	Chronic Stroke Group (*n* = 8)	*p* Value
Age, y/o, mean (SD)	51.5 (9.0)	52.3 (9.7)	0.77
Height, cm, mean (SD)	165.3 (5.9)	168.5 (9.1)	0.41
Weight, kg, mean (SD)	67.5 (10.2)	72.6 (16.9)	0.36
BBS, mean (SD)	56.0 (0.0)	43.5 (4.1)	0.00 **

** *p* < 0.01. SD = standard deviation. BBS = Berg balance scale.

**Table 3:** Comparison of the acceleration data of the six testing postures between the healthy group and the stroke group.

	Healthy Group (*n* = 8)	Chronic Stroke Group (*n* = 8)	*p* Value
SWS with E/O, mean (SD)	0.003 (0.001)	0.003 (0.001)	0.49
SWS with E/C, mean (SD)	0.003 (0.001)	0.005 (0.002)	0.07
FTS with E/O, mean (SD)	0.003 (0.001)	0.004 (0.002)	0.12
FTS with E/C, mean (SD)	0.004 (0.001)	0.005 (0.003)	0.13
STS with E/O, mean (SD)	0.005 (0.002)	0.005 (0.003)	0.65
STS with E/C, mean (SD)	0.005 (0.002)	0.010 (0.010)	0.10

SD = standard deviation. Unit: m/s^2^. SWS = shoulder-width stance. FTS = feet-together stance. STS = semi-tandem stance. E/O = eyes opened. E/C = eyes closed.

**Table 4:** Comparison of the gyroscope data of the six testing postures between the healthy group and the stroke group.

	Healthy Group (*n* = 8)	Chronic Stroke Group (*n* = 8)	*p* value
SWS with E/O, mean (SD)	1.679 (0.913)	4.801 (4.356)	0.02 *
SWS with E/C, mean (SD)	2.115 (0.899)	8.405 (6.226)	0.00 **
FTS with E/O, mean (SD)	3.420 (1.279)	8.386 (6.365)	0.01 **
FTS with E/C, mean (SD)	5.468 (2.196)	11.726 (7.132)	0.03 *
STS with E/O, mean (SD)	6.837 (3.718)	14.251 (6.911)	0.03 *
STS with E/C, mean (SD)	11.424 (4.700)	26.663 (15.080)	0.01 **

* *p* < 0.05. ** *p* < 0.01. SD = standard deviation. Unit: degree. SWS = shoulder-width stance. FTS = feet-together stance. STS = semi-tandem stance. E/O = eyes opened. E/C = eyes closed.

**Table 5:** Relationship between the smartphone balance test result and the BBS score.

	ACC		GYR	
	PCC	*p* Value	PCC	*P* Value
SWS with E/O	−0.191	0.478	−0.705	0.002 **
SWS with E/C	−0.492	0.053	−0.805	0.000 **
FTS with E/O	−0.427	0.099	−0.700	0.003 **
FTS with E/C	−0.395	0.130	−0.752	0.001 **
STS with E/O	−0.096	0.723	−0.725	0.001 **
STS with E/C	−0.470	0.067	−0.694	0.003 **

** *p* < 0.01 PCC = Pearson’s correlation coefficient ACC = accelerometer GYR = gyroscope SWS = shoulder-width stance FTS = feet-together stance STS = semi-tandem stance E/O = eyes opened E/C = eyes closed.
